# Date Palm Extract (*Phoenix dactylifera*) Encapsulated into Palm Oil Nanolipid Carrier for Prospective Antibacterial Influence

**DOI:** 10.3390/plants12213670

**Published:** 2023-10-25

**Authors:** Heba S. Elsewedy, Tamer M. Shehata, Nashi K. Alqahtani, Hany Ezzat Khalil, Wafaa E. Soliman

**Affiliations:** 1 Department of Pharmaceutical Sciences, College of Clinical Pharmacy, King Faisal University, Alhofuf 31982, Al-Ahsa, Saudi Arabia; 2 Department of Pharmaceutical Sciences, College of Pharmacy, Almaarefa University, Dariyah, Riyadh 13713, Saudi Arabia; 3 Department of Pharmaceutics, College of Pharmacy, Zagazig University, Zagazig 44519, Egypt; 4 Department of Food Science and Technology, College of Agriculture, King Faisal University, Alhofuf 31982, Al-Ahsa, Saudi Arabia; 5 Date Palm Research Center of Excellence, King Faisal University, Alhofuf 31982, Al-Ahsa, Saudi Arabia; 6 Department of Pharmacognosy, Faculty of Pharmacy, Minia University, Minia 61519, Egypt; 7 Department of Biomedical Sciences, College of Clinical Pharmacy, King Faisal University, Alhofuf 36362, Al-Ahsa, Saudi Arabia; 8 Department of Microbiology and Immunology, Faculty of Pharmacy, Delta University for Science and Technology, Mansoura 11152, Egypt

**Keywords:** palm oil, date palm extract, nanolipid carrier, natural products, optimization, antibacterial, topical drug delivery

## Abstract

It is worthwhile to note that using natural products today has shown to be an effective strategy for attaining the therapeutic goal with the highest impact and the fewest drawbacks. In Saudi Arabia, date palm (Phoenix dactylifera) is considered the principal fruit owing to its abundance and incredible nutritional benefits in fighting various diseases. The main objective of the study is to exploit the natural products as well as the nanotechnology approach to obtain great benefits in managing disorders. The present investigation focused on using the powder form of date palm extract (DPE) of Khalas cultivar and incorporates it into a nanolipid formulation such as a nanostructured lipid carrier (NLC) prepared with palm oil. Using the quality by design (QbD) methodology, the most optimized formula was chosen based on the number of assigned parameters. For more appropriate topical application, the optimized DP-NLC was combined with a pre-formulated hydrogel base forming the DP-NLC-hydrogel. The developed DP-NLC-hydrogel was evaluated for various physical properties including pH, viscosity, spreadability, and extrudability. Additionally, the in vitro release of the formulation as well as its stability upon storage under two different conditions of room temperature and refrigerator were investigated. Eventually, different bacterial strains were utilized to test the antibacterial efficacy of the developed formulation. The optimized DP-NLC showed proper particle size (266.9 nm) and in vitro release 77.9%. The prepared DP-NLC-hydrogel showed acceptable physical properties for topical formulation, mainly, pH 6.05, viscosity 9410 cP, spreadability 57.6 mm, extrudability 84.5 (g/cm^2^), and in vitro release 42.4%. Following three months storage under two distinct conditions, the formula exhibited good stability. Finally, the antibacterial activity of the developed DP-NLC-hydrogel was evaluated and proved to be efficient against various bacterial strains.

## 1. Introduction

The topical drug delivery system is a manner of applying the medication directly to the affected area, which is typically the skin. This method of administering medication has a number of advantages as an alternative to the most popular route of administration, the oral route [1]. Topical medication could avoid the problem of swallowing and lower bioavailability of the drug as a result of exposure to first pass metabolism [2]. Moreover, it evades gastrointestinal side effects providing more improvement in a patient’s recovery process [3]. In fact, topical preparations came in a variety of widely used formulations, including creams, ointments, lotions, and gels [4]. However, innovation in topical delivery has been adopted in recent years in order to offer more effective treatment and a remarkable future in health care.

Nanotechnology is one of the impressive techniques for developing drugs in a nanoscale form and overcoming the limitations of the majority of currently available medications [5]. It is considered as being a revolution in drug development where different nanocarriers can be formulated incorporating a variety of drugs with different characteristics for attaining more efficient formulations with respect to their design, structure, and activity [6]. Nanolipid carriers are widely used nanoformulations since they could overcome the solubility problem of the active ingredients and accommodate all kinds of drugs [7]. Additionally, the reason behind using nanolipid formulations in topical delivery is their ability to overcome the skin barrier [8]. The effectiveness of several nanolipid formulations such as anticancer, anti-inflammatory, antioxidants, antibacterial, and other agents has been demonstrated [9,10]. Moreover, and according to recent studies, antibacterial drugs work more effectively when they are released using drug delivery systems [11]. The nanostructured lipid carrier (NLC) is one of the nanolipid formulations that exhibited a great advance in the strategy of topical drug delivery [12]. The lipid phase in the NLC involves both liquid lipid (oil) and solid lipid (fat), which paved the way to incorporate hydrophobic drugs into the solid lipid part [7]. Consequently, a higher loading capacity could be obtained in addition to enhanced drug entrapment if compared to other nanolipid formulations [13]. However, using higher viscosity formulations would be preferable for effective topical application in order to extend their contact duration to the affected area. Consequently, in the majority of cases, the created nanolipid formulations were included into a pre-formulated hydrogel. 

Rationally, to obtain great efficiency and safety while using medication, natural compounds are the best to be utilized in drug development [14]. The majority of natural compounds are thought to be primarily found in plants. Date palm is a primary natural fruit spreading in arid regions such as Saudi Arabia that possess extreme nutritional and therapeutic values [15]. Moreover, it emphasized a great influence on being anticancer, anti-inflammatory, antifungal, and antibacterial [16,17]. A wide range of date cultivars are available comprising Sheshi, Reziz, and Khalas, which are broadly accessible in the Eastern Province of Saudi Arabia [18]. In fact, the benefits of date palm are uncountable because it contains numerous nutrients, including carbohydrates, vitamins, and minerals [19]. It could be used for treating most cardiovascular and hepatic disorders [20]. Furthermore, it is considered to be a good source of fiber and disease-fighting antioxidants, such as phytochemicals. The flavonoids, phenolic acids, and carotenoids contained in date palm are the most significant and advantageous phytochemicals [21]. Therefore, date palm is considered as a very effective tool for managing infectious diseases [22]. Previous literature emphasized the antibacterial activity of date palm extract against Gram-positive and Gram-negative bacteria [22,23]. For enhancing the activity of a certain compound, a combination could be applied with other products to provide a synergistic action. One significant vegetable oil that contains several biologically active components, such as fatty acids, is date oil. These fatty acids are present in the form of medium-chain triglycerides that converted into free fatty acids upon hydrolysis, which would provide an antibacterial effect [24]. In line with our previously reported estimation of total phenolic and flavonoid contents [17] and based on the abundance of such contents in the fruits of the Khalas variety date palm, it is expected to show good antibacterial activity; previous reports also demonstrated its effectiveness for such activity [25]. With the vision of an improvement in the route of application of date palm extract, the current work was designed to develop a unique nanolipid carrier formula.

Quality by design (QbD) methodology could be used to develop an optimized formula with the best qualities in order to maximize the benefits of the formulation [26]. Central composite design (CCD) is one of the important design expert software that could be used. It depends on detecting the influence of certain independent variables on selected dependent responses. 

In pursuit of this goal, our investigation was implemented in order to make use of the available natural products in addition to applying the concept of nanotechnology that was documented to show the extensive influence in treating various disorders. It depends greatly on studying the influence of natural products in managing skin disorders, mainly the bacterial infections. Additionally, the study tried to spot the light on the synergistic action that could be found between different natural products and help in improving the antibacterial effect. Therefore, DPE was incorporated into the NLC using palm oil (PO), and through CCD, the optimized formula was selected. As far as we know, this is the first investigation to study the influence of both DPE and PO in the same nanocarrier for antibacterial behavior. DP-NLC was incorporated into the hydrogel to be more appropriate for topical application. The physical properties of the developed NLC were evaluated followed by the antibacterial examination. 

## 2. Results

Fortunately, the attempt to obtain DPE was efficiently conducted and used in preparing various nanolipid formulations, mainly the NLC, using design expert software (CCD).

### 2.1. Central Composite Experimental Design

CCD is a tool for proposing different formulations of the NLC to be prepared depending on some independent variables and their influence on the response variables as Table 1 displays. The software proposed 11 NLC formulations that were analyzed by various methods. The model F-value was found to be 626.32 and 483.21 for X_1_ and X_2_, respectively, revealing a significant model. Moreover, the *p*-value was shown to be less than 0.05 in most of the model terms, which pointed toward significant model terms as well. Additionally, lack of fit is one more factor to be considered since it suggests the model fit [27]. It is essential for the lack of fit to be non-significant. As per the obtained results, lack of fit was 0.9409 and 0.3939 with *p*-values 0.5522 and 0.8411 for Y_1_ and Y_2_, respectively, which highlighted a non-significant lack of fit. 

### 2.2. Characterization of the Developed DP-NLCs

#### 2.2.1. Effect of Independent Variables X_1_ and X_2_ on Y_1_ Response

Table 1 presents data showing that the particle size of all developed NLC preparations ranged between 176 ± 3.5 and 337 ± 3.6 nm. This variation in particle size owed to the amount of each independent variable in the formulation. It could be observed that the presence of a high concentration of lipid phase in the formula would result in a greater particle size while using a lower lipid phase would provide the NLC with a smaller particle size. This fact could be ascribed to an increase in the dispersed phase [28]. On the other hand, regarding variable X_2_, it was noticeable that the presence of a higher concentration of surfactant while keeping the lipid phase concentration constant would result in an NLC with a smaller particle size. On the contrary, using a lower concentration of surfactant, while keeping the same concentration of lipid phase, would provide a larger size. This finding was explained based on surface tension since a higher surfactant concentration would lower the surface tension of the preparation, giving more stabilization for the surface and consequently, smaller particles [29,30]. The results were further approved by definite mathematical equations settled by CCD. This equation confirmed the direct relation between the lipid phase concentration and the particle size in addition to the inverse one between surfactant concentration and the particle size as is obvious in the following equation:

Y_1_= 262.263 + 64.3333 × X_1_ − 17.6667 × X_2_ + 1 × X_1_ X_2_ − 13.1579 × X_1_^2^ − 0.157895 × X_2_^2^ (1)

From Equation (1), it is clear that there is a positive sign in front of X_1_ that confirms a synergistic action between X_1_ and Y_1_ that would increase the particle size. Conversely, the negative sign in front of X_2_ was noticed, which characterized the antagonistic effect between X_2_ and Y_1_ where increasing X_2_ would diminish the particle size. 

**Figure 1:**
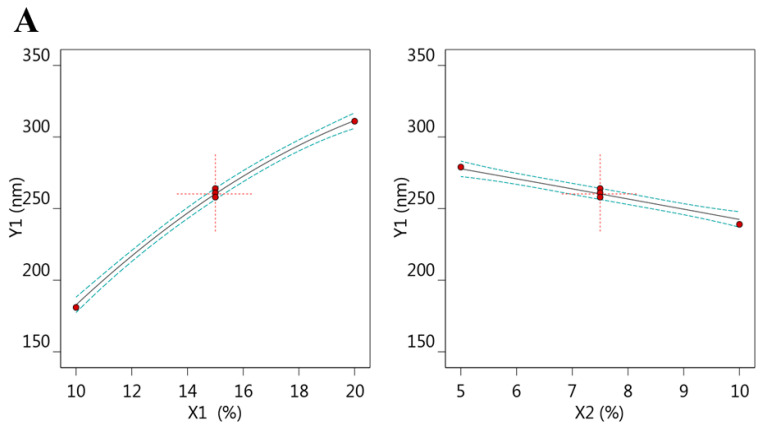

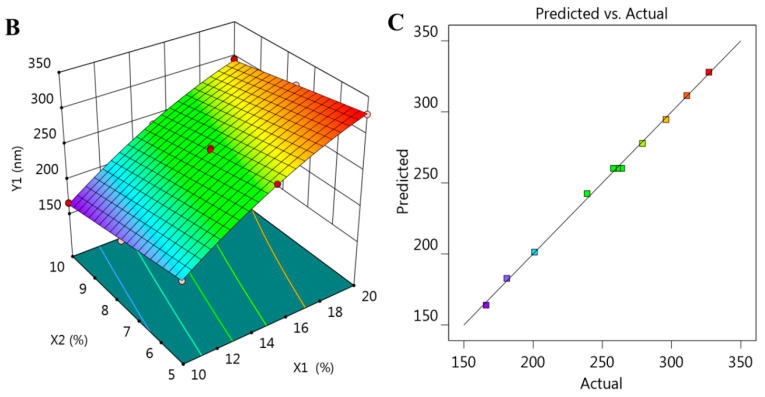
(**A**) All factor plot, (**B**) Three-dimensional response surface plot, and (**C**) Linear correlation plot between predicted against actual values for establishing the influence of variables X_1_ and X_2_ on the particle size response (Y_1_).

In addition, the design could create various graphs that would help in more illustration and confirmation of the results. Some of these graphs were all factor graphs and a three-dimensional response surface plot as Figure 1A,B depicts, respectively. As well, Figure 1C and Table 2 exhibit a correlation between adjusted and predicted R^2^ values that were 0.9968 and 0.9900, respectively, for the Y_1_ response. It was certified that these values were in reasonable agreement with each other as the difference between them is less than 0.2. Furthermore, the adequate precision was found to be (75.3680), which indicates an adequate signal that can be used to navigate the design space.

#### 2.2.2. Effect of Independent Variables X_1_ and X_2_ on Y_2_ Response

The amount of DP encapsulated into the NLC formulation was estimated via evaluating the percentage of EE. As Table 1 shows, the % of EE for all developed preparations was ranged among 60 ± 3.0 to 93 ± 3.3%. Focusing on the obtained findings, it could be concluded that a higher encapsulation of DP was achieved while using a higher concentration of lipid phase. The reason behind that came from the larger particle size that was achieved while using a higher lipid phase concentration, which could afford a larger amount of the drug due to the larger space available. On the opposite side, we could find that a smaller percentage of the drug was encapsulated into the formulation while using a lower lipid phase concentration, since the lipid space available was limited and consequently, could accommodate for only a small amount of the drug [31]. Additionally, the lipophilic nature of DP could help in encapsulating a larger amount of the drug as long as there is a large lipophilic space [32]. 

Additionally, and as mentioned above, our data could be emphasized by various ways proposed by the design expert. The attained mathematical equation could prove the positive direct relation between the lipid phase concentration and the resultant EE, which appeared in the positive sign in front of the X_1_ variable. However, there was a negative sign for the X_2_ variable indicating an indirect, antagonistic relation between the variable and response as Equation (2) shows:

Y_2_ = 75.7273 + 14 × X_1_ − 3 × X_2_ (2)

**Figure 2:**
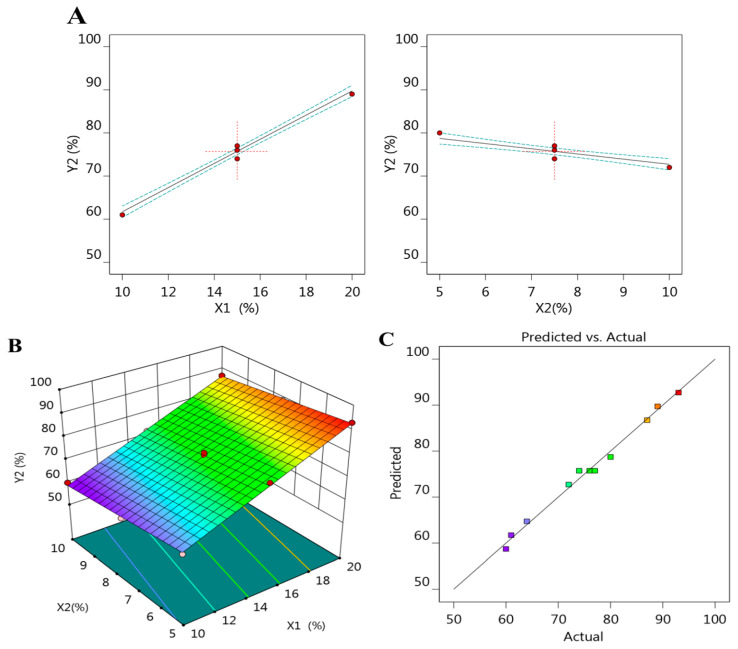
(**A**) All factor plot, (**B**) Three-dimensional response surface plot, and (**C**) Linear correlation plot between predicted versus actual values for illustrating the influence of variables X_1_ and X_2_ on the EE response (Y_2_).

Further, demonstrative graphs were constructed by the design that helps in emphasizing the results through an all factor graph and a three-dimensional response surface plot as Figure 2A,B show, respectively.

In addition to the aforementioned, and Table 2 and Figure 2C show, a linear correlation was seen between the adjusted and predicted R^2^ values that were 0.9897 and 0.9852, respectively. Added to that, the value of adequate precision was 57.7095 specifying an adequate signal and agreeing that the model can be used to navigate the design space.

### 2.3. Optimization Process

**Figure 3:**
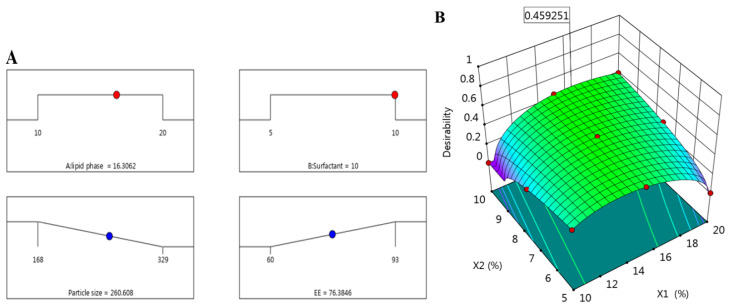
(**A**) Optimization ramp screening independent variable concentration, along with their expected values of responses; particle size and encapsulation efficiency, and (**B**) three-dimensional desirability figure illustrating the influence of X_1_ and X_2_ concentration on overall responses.

**Figure 4:**
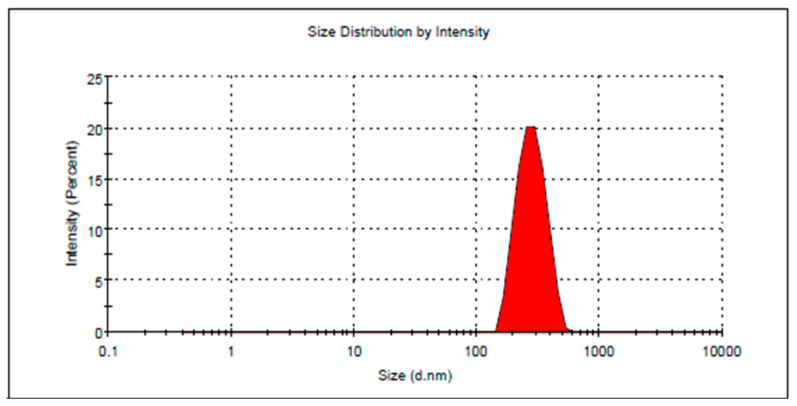
Particle size of optimized DP-NLC formulation.

In order to obtain an optimum formulation with required characteristics, the optimization process was employed via CCD using the point prediction option. In this process, all the dependent and independent variables were adjusted toward certain required criteria to attain the selected ideal formulation. In our study, and as Table 3 shows, the independent variables X_1_ and X_2_ were adjusted to be in range while both dependent variables were oriented to minimize Y_1_ and maximize Y_2_. According to the higher desirability value, the optimized formula was selected. The optimization process came up with the proposed concentration of X_1_ and X_2_ to be 16.3% and 10%, respectively, while the expected variables for both Y_1_ and Y_2_ to be 260.59 nm and 76.38%, respectively. As per these suggestions, the anticipated formulation was fabricated and assessed for the perceived responses. Noticeably, the observed data were very close to the predicted one as Table 3 and Figure 3 exhibit. In addition, Figure 4 presents the distribution curve of the optimized DP-NLC preparation as obtained in the experimental result of the particle size (266.9 ± 3.99) nm.

As stated above, viscous topical formulations are better and more efficient. Accordingly, the optimized DP-NLC was integrated into a pre-formulated HPMC hydrogel in order to obtain a novel DP-NLC-hydrogel. This newly developed formulation was subjected to a number of studies in order to check its effectiveness as a topical antibacterial formulation.

### 2.4. Topical Hydrogel Characterization

#### 2.4.1. Organoleptic Evaluation

Based on the physical examination of the prepared topical DP-NLC-hydrogel, it was obvious that the formula was well formulated. It showed a satisfactory physical appearance with a homogenous, smooth, and consistent texture as shown in Figure S1. 

#### 2.4.2. pH Value

It is renowned that topical preparations should possess pH very close to that of skin in the range of 4–6 to be calmative, soothing and nonirritant [33]. Referring to the data in Table 4, pH of the fabricated DP-NLC-hydrogel was perfect and could evade skin irritation. 

#### 2.4.3. Viscosity

As formerly discussed, appropriate viscosity of topical preparations is required for an easier and efficient application over the skin. Moreover, and according to the pharmacopoeial regulations, the viscosity of the topical formulations should be monitored [34]. Therefore, the viscosity of the DP-NLC-hydrogel was evaluated and established to be adequate for topical applications as Table 4 displays.

#### 2.4.4. Spreadability

Likewise, spreadability is an essential criterion that needed to be evaluated for topical preparations [35]. The lower the spreadability value, the more easier it is for the topical preparation to be extended over the skin [36]. The spreadability of the DP-NLC-hydrogel was in an acceptable range to be smeared over the skin as Table 4 reveals. 

#### 2.4.5. Extrudability

Extrudability is one of the essential parameters that should be taken into account and needed to be assessed for semisolid preparations to ensure patient compliance [37]. As Table 4 shows, the produced DP-NLC-hydrogel exhibited an ideal extrudability, which is convenient for the topical treatment. 

### 2.5. Studying In Vitro Release from DP-NLC-Hydrogel

**Figure 5:**
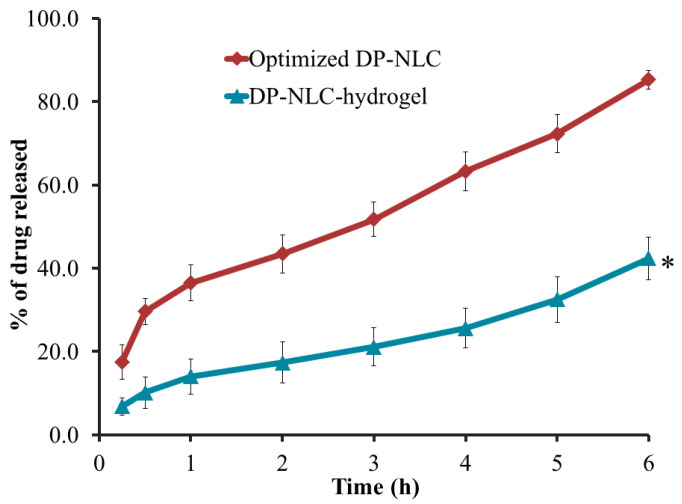
In vitro release of DP from optimized NLC and NLC-hydrogel formulation in phosphate buffer pH 5.5 at 32 °C ± 0.5. Results are shown as mean ± SD (n = 3). * *p* < 0.05 compared to DP-NLC formulation.

As Figure 5 depicts, the in vitro release of DP from various formulations including the DP-NLC-hydrogel and the optimized DP-NLC formulations was estimated. The study was carried out over 6 h providing a significant difference between the release of DP from the optimized NLC formulation (85.3 ± 2.3%) and that from the NLC-hydrogel (42.4 ± 5.2%) (*p* < 0.05). The reason behind the lower release from the hydrogel formulation when compared to the optimized NLC could be ascribed to the presence of a gelling agent in the preparation. Truthfully, the existence of a gelling agent would considerably affect the in vitro release since it provides a higher viscosity for the topical formulation [38]. The higher the viscosity, the lower the percentage of drug released from the preparation [39]. Although the in vitro release from the optimized DP-NLC seems to be more than that from the DP-NLC-hydrogel, nevertheless, the topical hydrogel reflected to be more desirable due to its proper viscosity and spreadability. These appropriate characters would result in more patient compliance as a result of an enhanced and easier topical application [40].

### 2.6. Stability Study

**Figure 6:**
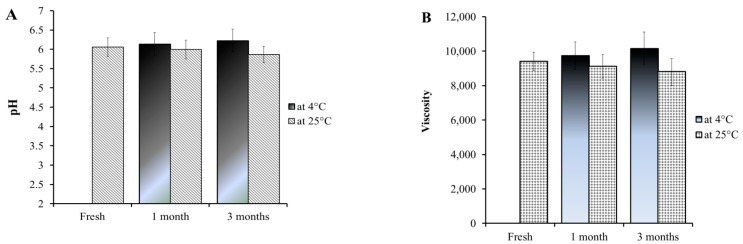

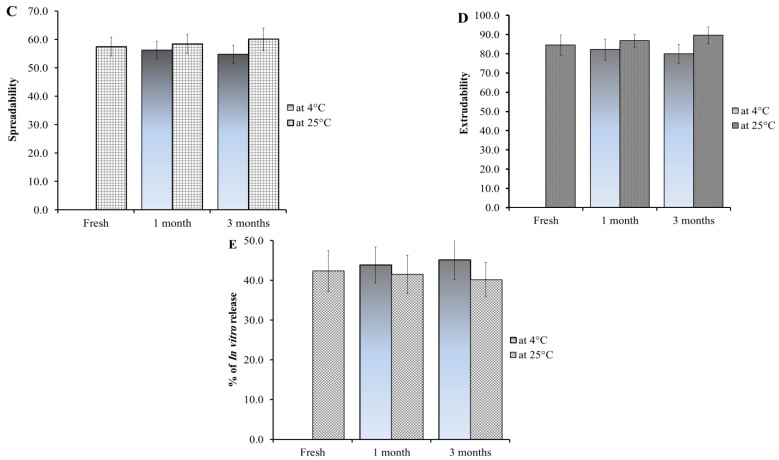
Stability profile of DP-NLC-hydrogel formulation following storage for 1 and 3 months at 4 °C and 25 °C relative to (**A**) pH, (**B**) viscosity, (**C**) spreadability, (**D**) extrudability, and (**E**) % of in vitro drug release compared to same formulation while fresh.

The stability test is very important in pharmaceutical manufacturing for determining the optimum storage conditions [41]. In the current investigation, stability testing was employed for the prepared DP-NLC-hydrogel compared to the same formulation while fresh, following storage for 3 months at two conditions. As Figure 6 shows, for all estimated parameters, comprising pH, viscosity, spreadability, and extrudability, non-significant changes were detected for the whole period of storage (*p* < 0.5). The obtained findings established that the DP-NLC-hydrogel exhibited good stability, which ensures the proficiency of the NLC-hydrogel as a nanocarrier for delivering different active compounds [42]. Moreover, the result emphasized the considerable stability of the NLC formulation owing to its structure as a lipid base shielded with surfactant [43].

### 2.7. Skin Irritation Test

It is very important to ensure the safety of a topical preparation when applied topically over the skin. Therefore, a skin irritation test is a very essential experiment to be performed for sensitivity reactions that might happen. Based on that, the developed DP-NLC-hydrogel was applied on the back of the animals under investigation and it was observed that there were no marks of irritation, erythema, or edema on the treated area during the whole experiment [44]. This truly would confirm the safety of the formulation to be applied topically.

### 2.8. Antibacterial Study

**Figure 7:**
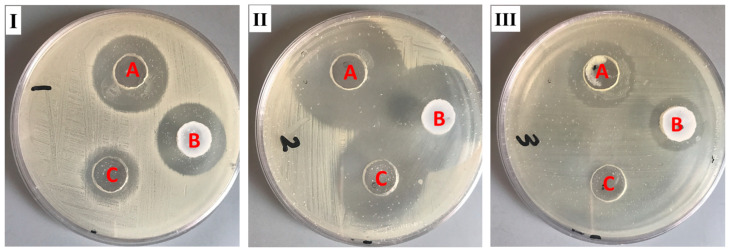
Zone of inhibition caused by the examined formulations. (A) DP-NLC-hydrogel, (B) FA cream (fucidin^®^), and (C) Blank NLC-hydrogel on different bacteria: (**I**) *Bacillus subtilis*, (**II**) *Staphylococcus aureus*, and (**III**) *klebsiella pneumoniae*.

For evaluating the antibacterial behavior of the DP-NLC-hydrogel, such an investigation was implemented against different bacterial strains. This is simply performed by assessing the inhibition zone that was initiated owing to the action of the formulation alongside the bacteria. As Table 5 and Figure 7 reveal, the inhibition zone was assessed for the DP-NLC-hydrogel, blank NLC-hydrogel, and marketed FA (fucidin^®^). It was noticeable that the DP-NLC-hydrogel was very active against different bacteria: Bacillus subtilis, Staphylococcus aureus, and klebsiella pneumoniae, since it exhibited a significant inhibition zone when compared to the blank NLC-hydrogel *p* < 0.05. However, there was a non-significant difference obtained between the DP-NLC-hydrogel and the marketed product (*p* < 0.05) in cases of Bacillus subtilis and Staphylococcus aureus, which fortunately emphasized the activity of the developed formulation in inhibiting the bacterial growth as well as the marketed product. In contrast, a significant difference was obtained for klebsiella pneumoniae when compared with marketed formulae (*p* < 0.05). Interestingly, investigating the antibacterial activity of the blank NLC-hydrogel revealed a remarkable inhibition for the bacterial growth in the media, though it has no DP, which is presumed to be due to the presence of palm oil in the formulation. This result highlighted the documented fact about the antibacterial behavior of palm oil [45]. The reason behind its antibacterial activity is the presence of saturated and unsaturated fatty acids, mainly, the saturated myristic acid with broad antioxidant, anticancer, and antibacterial activity [46,47]. Conclusively, the extensive antibacterial activity observed by the DP-NLC-hydrogel could be accredited to the presence of DP and palm oil in the same formulation.

### 2.9. Morphology of Treated Bacterial Cells

**Figure 8:**
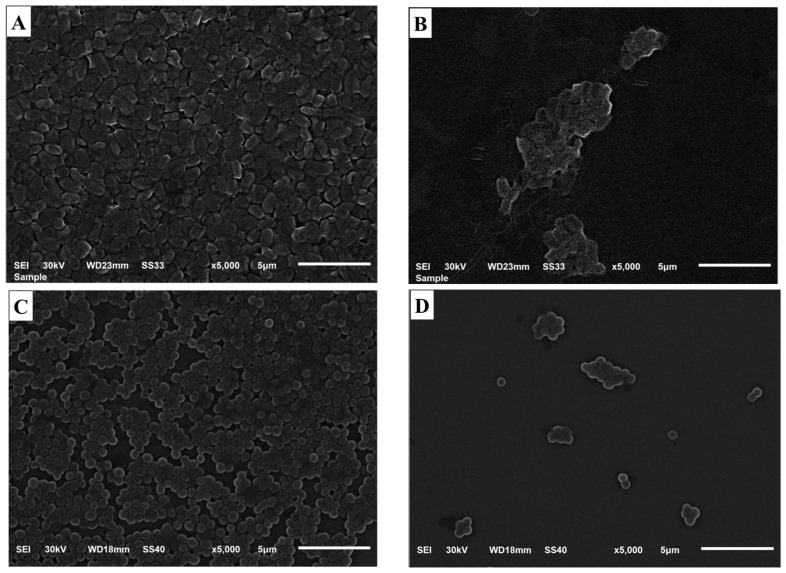
SEM images displaying the pattern of the bacterial biofilms formed following 24 h of incubation and its inhibition. (**A**) Control Klebsiella pneumoniae, (**B**) Klebsiella pneumoniae treated with DP-NLC-hydrogel, (**C**) Control staphylococcus aureus, and (**D**) Staphylococcus aureus treated with DP-NLC-hydrogel.

The morphology of different bacterial cells treated with the DP-NLC-hydrogel formulation was performed utilizing SEM and Figure 8 reveals the results. It is highly demonstrated from the images that there are more cells adhered to the surface of the control Staphylococcus aureus and klebsiella pneumoniae bacterial cells (Figure 8A and Figure 6C) compared to the treated cells (Figure 8B and Figure 6D). It was revealed that there is a great reduction in the number of bacterial cells following treatment with the DP-NLC-hydrogel formulation as this appeared in the extensive change in the bacterial morphology, which revealed the efficiency of the developed formulation as an antibacterial agent. The results were in accordance with Qasim et al. who noticed the inhibition of the bacterial growth while using Khalas extract [48].

## 3. Materials and Methods

### 3.1. Material

Palm oil, stearic acid, Tween 80, and Hydroxpropyl methylcellulose (HPMC) were obtained from Sigma Aldrich (St. Louis, MO, USA). MC60 glycerol monocaprylocaprate (Labrafac™) was procured from Gattefosse SAS (Saint-priest Cedex-France). All other reagents were of the finest grade available.

### 3.2. Manufacture of Date Palm Extract (DPE)

#### 3.2.1. Identification and Assembly of Dates

Fruits of Khalas cultivar date palm (the palatable stage) were purchased from the local markets in Al-Ahsa, Eastern Province of Saudi Arabia. The fruits were identified by experts and taxonomists in the Date Palm Research Center, King Faisal University, Al-Ahsa, Saudi Arabia. A voucher specimen of the fruit Khalas date palm was deposited in the Herbarium of the Department of Pharmaceutical Sciences, College of Clinical Pharmacy, King Faisal University, Al-Ahsa, Saudi Arabia (20-Sept-KH) [17].

#### 3.2.2. Formulation of Crude Extract

A previously prepared date palm methanol extract (DPE) [17] with a weight of 250 g of dried flesh of fruits of Khalas date palm was extracted using 2.5 L of 70% methanol in distilled water. Then the extract was concentrated under reduced pressure to give dried DPE that was kept in a dried form for further studies. A voucher specimen for the date palm of the fruit Khalas was placed in the Herbarium of Pharmaceutical Sciences Department, College of Clinical Pharmacy, King Faisal University, Al-Ahsa, Saudi Arabia (20-Sept-KH).

### 3.3. Central Composite Experimental Design

In the present investigation, CCD was employed as one of the most widely used factorial designs. In view of that, Design-Expert version 12.0 software (Stat-Ease, Minneapolis, MN, USA) was applied for carrying out the optimization process. Two-level, two-factor (22) factorial design was assembled in which two independent variables were investigated for their effect on two selected dependent responses. In the current design, the independent variables are lipid phase concentration (X_1_) and surfactant concentration (X_2_); whereas the dependent responses were particle size (Y_1_) and encapsulation efficiency (Y_2_). Using the analysis of variance test (ANOVA), all the statistical issues were checked in addition to some constructed modeling plots that help in illustrating the design such as the three-dimensional response surface graph and prediction versus actual plot. Moreover, polynomial mathematical equations were created to emphasize the obtained data and influences [26]. Table 6 shows the selected independent variables that were examined at two levels against two dependent responses.

### 3.4. Fabrication of DP-NLC

Eleven NLC formulations were prepared using DPE and PO via employing a design expert methodology, CCD, using the melt emulsification–ultrasonication method [49]. Based on the preliminary study performed, the best ratio between solid lipid and liquid lipid phase that was 2:8 was utilized. Basically, the definite amount of stearic acid (with melting point 69.3 °C) was subjected to a temperature higher than its melting point. Then, PO as a liquid lipid phase was added to the melted stearic acid, forming the lipid phase. DPE (175 mg) and the Labrafac™ co-surfactant (0.5 g) were added to the melted lipid phase with constant stirring until a uniform mixture was attained. For developing the emulsion, up to 10 mL of the aqueous phase was used to which a specified amount of surfactant (Tween 80) was added. The formed aqueous phase was heated at the same temperature first and then slowly added to the lipid phase while stirring. Using the Ultra-Turrax homogenizer (IKA-T25; Staufen, Germany), the acquired emulsion was homogenized for 5 min at 10,000 rpm followed by sonicating the mixture for 30 s via the probe sonicator XL-200, Qsnonica (Newtown, CT, USA) [50]. The formed dispersion was preserved at room temperature for cooling and assembly of the NLC.

### 3.5. Particle Size and Poly Dispersability Index (PDI) Analysis

For determining the particle size of the prepared DP-NLC and their corresponding PDI, Zetasizer apparatus (Malvern Instruments Ltd., Worcestershire, UK) was utilized. The measurements were accomplished using the dynamic light scattering technique, keeping the temperature at 25 °C [30].

### 3.6. Encapsulation Efficiency (EE)

A centrifugation method that was previously performed by Haroun et al., 2022 was applied for measuring the percentage of DP encapsulated into the formulated NLCs [51]. In brief, the DP-NLC sample was added into the centrifugal tube Amicon^®^ ultra-4 (Ultracel-10K) to be centrifuged at a rotation of 4000 rpm utilizing centrifuge (Andreas Hettich GmbH, Co.KG, Westphalia, Germany) for 1 h at a temperature of 4 °C. The filtrate containing the free drug was gathered, diluted, and then assayed via spectrophotometer (U.V. Spectrophotometer, JENWAY 6305, Bibby Scientific Ltd., Staffs, UK) at wavelength 261 nm. The percentage of EE was determined from the following equation:

% EE = [(Whole drug − Free drug)/Whole drug] × 100

### 3.7. Studying In Vitro Release

For assessing the efficacy and the quality of the NLC as a drug delivery system, an in vitro release study was executed [52]. The purpose of the investigation is to evaluate the percentage of the DP released from the fabricated NLC system. It is implemented using the ERWEKA dissolution system (ERWEKA, GmbH, Heusenstamm, Germany) where glass tubes were covered with a cellophane membrane (MWCO 2000–15,000) from one side and fixed to the apparatus from the other side. A sample of the DP-NLC was added to the test tube to be over the cellophane membrane. The tubes were dipped into the vehicle that consists of phosphate buffer pH 5.5 and represents the release media. The device was operated and adjusted to rotate at 50 rpm with the temperature kept at 32 ± 0.5 °C. The release was continued for 6 h and a sample was examined for DP, released at a selected interval of time. Each withdrawn sample was replaced with an equivalent amount of fresh vehicle [26]. The withdrawn sample was assayed through a spectrophotometer (JENWAY 6305, Bibby Scientific Ltd., Staffs, UK) at λ_max_ 261 nm to check the absorbance. The study was carried out in triplicate.

### 3.8. Fabrication of Topical Formulation

Evidently, the NLC was used extensively for topical administration to treat various skin disorders [53]. However, its low viscosity, if compared to other topical preparations, was considered to be a hurdle in providing longer contact time with the affected area and consequently, it would affect the efficiency. In view of that, increasing the viscosity of the formulation would be appropriate since the formula would spread uniformly and regularly over the affected skin [54]. Therefore, a more viscous topical preparation was developed via incorporating the optimized DP-NLC into a pre-formulated hydrogel. Primarily, the hydrogel was prepared using a suitable gelling agent, namely, 4% *w*/*w* of HPMC that was scattered into 15 mL distilled water while stirring to obtain HPMC-hydrogel using a magnetic stirrer (Jeio Tech TM-14SB, Medline Scientific, Oxfordshire, UK). Next, the prepared hydrogel was mixed with the optimized DP-NLC using a mixer (Heidolph RZR 1, Heidolph Instruments, Schwabach, Germany) for 5 min in order to obtain the DP-NLC-hydrogel. Following the same method, a blank NLC-hydrogel was developed, free from DP, for checking the effectiveness of the DP-NLC-hydrogel.

### 3.9. Topical Hydrogel Characterization

#### 3.9.1. Organoleptic Evaluation

DP-NLC-hydrogel was examined visually for its physical characteristics including appearance, color, and homogeneity and was then checked for any probable agglomeration or phase separation [55].

#### 3.9.2. pH Value

It is essential for pH of formulations intended for topical application to be very close to skin pH in order to not be an irritant. Accordingly, the pH of the DP-NLC-hydrogel was assessed utilizing a standardized pH meter (MW802, Milwaukee Instruments, Szeged, Hungary) by dipping the glass electrode of the pH meter into a sufficient amount of the formulation [56].

#### 3.9.3. Viscosity

As previously mentioned, viscosity is a very crucial parameter to be measured. Therefore, viscosity of the DP-NLC-hydrogel was evaluated at 25 °C utilizing a Brookfield viscometer (DV-II+ Pro, Middleboro, MA, USA) using spindle R5, and adjusting the rotation at 0.5 rpm [10].

#### 3.9.4. Spreadability

Since an even application of the formulation over the skin is very important, thus spreadability of the DP-NLC-hydrogel was determined; 1 gm of the formulation was put in between two slides made of glass (25 cm × 25 cm) and then a load of 500 mg was fixed for 1 min over the system. The formulation was spread on the slide and the diameter was measured which indicated the spreadability of the preparation over the skin area [14].

#### 3.9.5. Extrudability

The ability of any topical formulation to be extruded out of a collapsed tube in the form of a ribbon is known as extrudability. In this examination, the weight in grams was calculated that allowed at least half a centimeter of ribbon of the hydrogel to be extruded out upon pressing the crimped end of the collapsed tube [57,58]. The extrudability (g/cm^2^) was calculated using the following equation:

Extrudability = Weight applied to extrude topical preparation out of tube (g)/Area (cm^2^)

### 3.10. Studying In Vitro Release from DP-NLC-Hydrogel

The same technique was implemented in determining the in vitro release from the optimized DP-NLC and was followed in order to determine the in vitro release from the developed DP-NLC-hydrogel. The process was carried out by operating the ERWEKA dissolution system (ERWEKA, GmbH, Heusenstamm, Germany).

### 3.11. Stability Test

Studying the stability of the formulation provides very substantial proof on how the characterization of the developed formulation might change with time when kept under certain adjusted conditions. The DP-NLC-hydrogel was examined for various parameters including physical appearance, pH, viscosity, and spreadability, in addition to the in vitro release study. The investigation was executed in accord with the International Conference on Harmonization (ICH) guidelines after keeping the formulation under two different circumstances: 4 ± 2 °C and at 25 ± 2 °C for 1 and 3 months. The formulation was examined and compared to the same preparation while fresh [59].

### 3.12. Animals

#### 3.12.1. Experimental Design for Animals

The current study was carried out using male Wister rats, each of about 220–250 g. The rats were supplied from the Experimental Animal Research Centre at King Saud University, Riyadh, KSA. All rats were accommodated under suitable housing conditions at ambient temperature (25 ± 2 °C) and a light/dark cycle (12:12 h) with free access to water.

#### 3.12.2. Approval of Ethical Code

The protocols of animal work were executed following the Institutional Animal Care and Use Committee approval. The investigations were in accordance with the guidelines of the Research Ethics Committee (REC) at King Faisal University, approval number (KFU-REC/2022-May–ETHICS17).

#### 3.12.3. Skin Irritation Test

The best topical preparation is the one that shows no sign of irritation upon application. Consequently, the developed DP-NLC-hydrogel should be examined for its safety upon spreading over the skin via performing a skin irritation test. Simply, the rats proposed for the study were prepared by shaving the dorsal hair by electric clipper the day before starting the experiment. On the shaved area, the developed DP-NLC-hydrogel was spread evenly and kept for 7 days to be observed for any sign of irritation, erythema, or edema. There is a sensitivity scale which ranges between 0 and 3 that helps in detecting the severity of the condition, if any, where 0 indicates no irritation while 3 suggests severe erythema with or without edema [60].

### 3.13. Antibacterial Study

The influence of the investigated formulations toward bacterial growth was examined using various bacterial strains via disc diffusion method [26,61]. These bacterial strains were *Bacillus subtilus* (ATCC 10400), *Staphylococcus aureus* (ATCC 29213), and *Klebsiella pneumoniae* (ATCC 10013) that were obtained from the American Type Culture Collection (ATCC). In short, Mueller–Hinton Agar was prepared in a Petri dish and disseminated to form the culture media for the bacteria. In each Petri, there were 3 wells each of 6 mm diameter to be filled with the examined formulation. The DP-NLC-hydrogel, blank NLC-hydrogel, and marketed antibacterial preparation (fucidin^®^) were investigated by pouring into wells and incubated for 24 h at 37 ± 1 °C. The diameter of the inhibition zone was measured and provided a suggestion for the antibacterial activity of the formulation [62]. Each experiment was conducted in triplicate with mean value ± SD.

### 3.14. Morphology of Treated Bacterial Cells

For confirming the antibacterial influence of the DP-NLC-hydrogel, the morphology of the bacterial strains before and after the treatment was studied using scanning electron microscopy. To sum up, a mixture of the DP-NLC-hydrogel (100 μg), Mueller–Hinton broth (100 μL), and 10 μL microbe (about 1.5 × 106 CFU/mL) was prepared and incubated for 1 h. After that, the mixture was exposed to centrifugation at 15,000 rpm for 20 min to remove the supernatant and keep the pellets suspended in a normal saline. An aliquot of about 50 μL of the suspension was distributed over the slide and kept to dry. The sample was fixed in 3% glutaraldehyde for 3 h and then scrutinized for its morphology utilizing a scanning electron microscope. The scanning electron microscope was also implemented for the bacteria before treatment, which was considered as a control [63].

### 3.15. Statistical Analysis

Data were expressed as mean ± SD with each study performed at least three times. The *p* value < 0.05 was considered statistically significant using the one-way analysis of variance (ANOVA) test. SPSS statistics software was applied for detecting the statistical analysis, version 9 (IBM Corporation, Armonk, NY, USA).

## 4. Conclusions

Employing quality by a design approach was helpful for selecting the more optimized nanolipid formulation prepared with date palm extract, which is the most famous fruit in Saudi Arabia. The formula was prepared using a natural lipid phase such as palm oil in order to maximize the benefits. The optimized date palm nanolipid formula was integrated into a pre-formulated hydrogel base for a potential topical effect. The developed DP-NLC-hydrogel was evaluated for its physical characteristics and proved to be ideal for topical application. It exhibited good stability over 3 months in storage under different conditions. Ultimately, it could result in a significant increase in the bacterial inhibition zone, which points toward a good antibacterial influence of DP-NLC-hydrogel. The result might highlight the influence of combining date palm extract and palm oil in improving the antibacterial activity of the formulation and verify the prospective impact for the nanolipid formula as a nanocarrier.

## Figures and Tables

**Table 1:** The experimental runs using the independent variables along with their observed dependent responses as per CCD.

Formula	Selected Independent Variables		Observed Responses	
	X_1_ (%)	X_2_ (%)	Y_1_ (nm)	Y_2_ (%)
F1	10	7.5	191 ± 2.6	61 ± 3.3
F2	10	10	176 ± 3.5	60 ± 3.0
F3	15	10	249 ± 4.5	72 ± 2.7
F4	10	5	211 ± 3.3	64 ± 2.5
F5	15	7.5	274 ± 4.1	74 ± 3.6
F6	15	7.5	271 ± 3.9	76 ± 3.1
F7	20	7.5	321 ± 3.6	89 ± 3.5
F8	15	5	289 ± 3.1	80 ± 3.9
F9	20	10	306 ± 3.6	87 ± 2.5
F10	20	5	337 ± 4.0	93 ± 3.3
F11	15	7.5	268 ± 3.1	77 ± 4.7

X_1_: lipid phase concentration; X_2_: surfactant concentration; Y_1_: particle size, and Y_2_: EE.

**Table 2:** Fit statistics.

R		
	Y_1_	Y_2_
R^2^	0.9984	0.9918
Adjusted R^2^	0.9968	0.9897
Predicted R^2^	0.9900	0.9852
Adequate precision	75.3680	57.7095

**Table 3:** Predicted and experimental results of the selected optimized DP-NLC formulation.

Independent Variable	Symbol	Constraint
Lipid phase concentration	X_1_	In range
Surfactant concentration	X_2_	In range
Response	Predicted values	Observed values
Y_1_ (nm)	260.59 ± 2.94	266.9 ± 3.99
Y_2_ (%)	76.38 ± 1.12	77.9 ± 2.2

**Table 4:** Topical characterization of DP-NLC-hydrogel.

Character	DP-NLC-Hydrogel
Homogeneity	Smooth and homogenous
pH	6.05 ± 0.25
Viscosity (cP)	9410 ± 708
Spreadability (mm)	57.6 ± 3.4
Extrudability (g/cm^2^)	84.5 ± 5.4

Data are expressed as mean ± (SD).

**Table 5:** Antibacterial activity of inspected formulations against different bacterial strains.

Bacterial Type	Inhibition Zone (cm)		
	DP-NLC-Hydrogel	Marketed Cream (fucidin^®^)	Blank NLC-Hydrogel
*Bacillus subtilis*	2.9 ± 0.17 *	2.73 ± 0.15 *	1.8 ± 0.10
*Staphylococcus aureus*	4.3 ± 0.13 *	4.1 ± 0.10 *	2.9 ± 0.11
*klebsiella pneumoniae*	2.4 ± 0.12 *	1.9 ± 0.10 *	0.9 ± 0.11

Values are DP-NLC-hydrogel expressed as mean ± SD. * (*p* < 0.05) compared to blank formulation.

**Table 6:** Selected independent variables and their level of variation in CCD.

Independent Variable	Level of Variation	
	(−1)	(+1)
Lipid phase concentration (%)	10	20
Surfactant concentration (%)	5	10

## Data Availability

Not applicable.

## Supplementary Materials

The following supporting information can be downloaded at: https://www.mdpi.com/article/10.3390/plants12213670/s1, Figure S1: Sample: is a nanostructured liquid carrier prepared with palm oil and containing Date palm extract. Control: is a nanostructured liquid carrier prepared with palm oil without Date palm extract.

## References

[1] Sastry S.V., Nyshadham J.R., Fix J.A. (2000). Recent technological advances in oral drug delivery-a review. Pharm. Sci. Technol. Today.

[2] Andrade F., Roca-Melendres M.M., Llaguno M., Hide D., Raurell I., Martell M., Vijayakumar S., Oliva M., Schwartz S., Durán-Lara E.F. (2022). Smart and eco-friendly N-isopropylacrylamide and cellulose hydrogels as a safe dual-drug local cancer therapy approach. Carbohydr. Polym..

[3] Tanner T., Marks R. (2008). Delivering drugs by the transdermal route: Review and comment. Ski. Res. Technol..

[4] Barnes T.M., Mijaljica D., Townley J.P., Spada F., Harrison I.P. (2021). Vehicles for Drug Delivery and Cosmetic Moisturizers: Review and Comparison. Pharmaceutics.

[5] Malik S., Muhammad K., Waheed Y. (2023). Nanotechnology: A Revolution in Modern Industry. Molecules.

[6] Pereira A., Valdés-Muñoz E., Marican A., Cabrera-Barjas G., Vijayakumar S., Valdés O., Rafael D., Andrade F., Abaca P., Bustos D. (2023). Rational Design of Hydrogels for Cationic Antimicrobial Peptide Delivery: A Molecular Modeling Approach. Pharmaceutics.

[7] Elsewedy H.S., Shehata T.M., Almostafa M.M., Soliman W.E. (2022). Hypolipidemic Activity of Olive Oil-Based Nanostructured Lipid Carrier Containing Atorvastatin. Nanomaterials.

[8] Hua S. (2015). Lipid-based nano-delivery systems for skin delivery of drugs and bioactives. Front. Pharmacol..

[9] Ferreira K.C.B., Valle A., Paes C.Q., Tavares G.D., Pittella F. (2021). Nanostructured Lipid Carriers for the Formulation of Topical Anti-Inflammatory Nanomedicines Based on Natural Substances. Pharmaceutics.

[10] Elsewedy H.S., Shehata T.M., Soliman W.E. (2022). Shea Butter Potentiates the Anti-Bacterial Activity of Fusidic Acid Incorporated into Solid Lipid Nanoparticle. Polymers.

[11] Carreño G., Marican A., Vijayakumar S., Valdés O., Cabrera-Barjas G., Castaño J., Durán-Lara E.F. (2020). Sustained Release of Linezolid from Prepared Hydrogels with Polyvinyl Alcohol and Aliphatic Dicarboxylic Acids of Variable Chain Lengths. Pharmaceutics.

[12] Elmowafy M., Al-Sanea M.M. (2021). Nanostructured lipid carriers (NLCs) as drug delivery platform: Advances in formulation and delivery strategies. Saudi Pharm. J..

[13] Patel D., Dasgupta S., Dey S., Ramani Y.R., Ray S., Mazumder B. (2012). Nanostructured Lipid Carriers (NLC)-Based Gel for the Topical Delivery of Aceclofenac: Preparation, Characterization, and In Vivo Evaluation. Sci. Pharm..

[14] Elsewedy H.S., Shehata T.M., Soliman W.E. (2022). Tea Tree Oil Nanoemulsion-Based Hydrogel Vehicle for Enhancing Topical Delivery of Neomycin. Life.

[15] Hamad I., AbdElgawad H., Al Jaouni S., Zinta G., Asard H., Hassan S., Hegab M., Hagagy N., Selim S. (2015). Metabolic Analysis of Various Date Palm Fruit (*Phoenix dactylifera* L.) Cultivars from Saudi Arabia to Assess Their Nutritional Quality. Molecules.

[16] Selim S., Alfy S., Al-Ruwaili M., Abdo A., Jaouni S. (2012). Susceptibility of imipenem-resistant Pseudomonas aeruginosa to flavonoid glycosides of date palm (*Phoenix dactylifera* L.) tamar growing in Al Madinah, Saudi Arabia. Afr. J. Biotechnol..

[17] Khalil H.E., Alqahtani N.K., Darrag H.M., Ibrahim H.-I.M., Emeka P.M., Badger-Emeka L.I., Matsunami K., Shehata T.M., Elsewedy H.S. (2021). Date palm extract (*Phoenix dactylifera*) PEGylated nanoemulsion: Development, optimization and cytotoxicity evaluation. Plants.

[18] Alhaider I.A., Mohamed M.E., Ahmed K., Kumar A.H. (2017). Date palm (*Phoenix dactylifera*) fruits as a potential cardioprotective agent: The role of circulating progenitor cells. Front. Pharmacol..

[19] Mattila P., Hellström J., Törrönen R. (2006). Phenolic acids in berries, fruits, and beverages. J. Agric. Food Chem..

[20] Tang Z.X., Shi L.E., Aleid S.M. (2013). Date fruit: Chemical composition, nutritional and medicinal values, products. J. Sci. Food Agric..

[21] Alfaro-Viquez E., Roling B.F., Krueger C.G., Rainey C.J., Reed J.D., Ricketts M.L. (2018). An extract from date palm fruit (*Phoenix dactylifera*) acts as a co-agonist ligand for the nuclear receptor FXR and differentially modulates FXR target-gene expression in vitro. PLoS ONE.

[22] Al-Shwyeh H.A. (2019). Date Palm (*Phoenix dactylifera* L.) Fruit as Potential Antioxidant and Antimicrobial Agents. J. Pharm. Bioallied Sci..

[23] Alrajhi M., Al-Rasheedi M., Eltom S.E.M., Alhazmi Y., Mustafa M.M., Ali A.M. (2019). Antibacterial activity of date palm cake extracts (*Phoenix dactylifera*). Cogent Food Agric..

[24] Hovorková P., Laloučková K., Skřivanová E. (2018). Determination of in vitro antibacterial activity of plant oils containing medium-chain fatty acids against gram-positive pathogenic and gut commensal bacteria. Czech J. Anim. Sci..

[25] Bhaskaracharya R.K., Bhaskaracharya A., Stathopoulos C. (2023). A systematic review of antibacterial activity of polyphenolic extract from date palm (*Phoenix dactylifera* L.) kernel. Front. Pharmacol..

[26] Almostafa M.M., Elsewedy H.S., Shehata T.M., Soliman W.E. (2022). Novel Formulation of Fusidic Acid Incorporated into a Myrrh-Oil-Based Nanoemulgel for the Enhancement of Skin Bacterial Infection Treatment. Gels.

[27] Carreño G., Pereira A., Ávila-Salas F., Marican A., Andrade F., Roca-Melendres M.M., Valdés O., Vijayakumar S., Schwartz S., Abasolo I. (2021). Development of “on-demand” thermo-responsive hydrogels for anti-cancer drugs sustained release: Rational design, in silico prediction and in vitro validation in colon cancer models. Mater. Sci. Eng. C.

[28] Sarheed O., Dibi M., Ramesh K.V. (2020). Studies on the effect of oil and surfactant on the formation of alginate-based O/W lidocaine nanocarriers using nanoemulsion template. Pharmaceutics.

[29] Chuacharoen T., Prasongsuk S., Sabliov C.M. (2019). Effect of Surfactant Concentrations on Physicochemical Properties and Functionality of Curcumin Nanoemulsions Under Conditions Relevant to Commercial Utilization. Molecules.

[30] Soliman W.E., Elsewedy H.S., Younis N.S., Shinu P., Elsawy L.E., Ramadan H.A. (2022). Evaluating Antimicrobial Activity and Wound Healing Effect of Rod-Shaped Nanoparticles. Polymers.

[31] Thatipamula R., Palem C., Gannu R., Mudragada S., Yamsani M. (2011). Formulation and in vitro characterization of domperidone loaded solid lipid nanoparticles and nanostructured lipid carriers. Daru J. Fac. Pharm. Tehran Univ. Med. Sci..

[32] Anwar W., Dawaba H.M., Afouna M.I., Samy A.M., Rashed M.H., Abdelaziz A.E. (2020). Enhancing the oral bioavailability of candesartan cilexetil loaded nanostructured lipid carriers: In vitro characterization and absorption in rats after oral administration. Pharmaceutics.

[33] Lukić M., Pantelić I., Savić S.D. (2021). Towards Optimal pH of the Skin and Topical Formulations: From the Current State of the Art to Tailored Products. Cosmetics.

[34] Chang R.K., Raw A., Lionberger R., Yu L. (2013). Generic development of topical dermatologic products: Formulation development, process development, and testing of topical dermatologic products. AAPS J..

[35] Djiobie Tchienou G.E., Tsatsop Tsague R.K., Mbam Pega T.F., Bama V., Bamseck A., Dongmo Sokeng S., Ngassoum M.B. (2018). Multi-Response Optimization in the Formulation of a Topical Cream from Natural Ingredients. Cosmetics.

[36] Rostami M., Farzaneh V., Boujmehrani A., Mohammadi M., Bakhshabadi H. (2014). Optimizing the extraction process of sesame seed’s oil using response surface method on the industrial scale. Ind. Crops Prod..

[37] Khan B.A., Ahmad S., Khan M.K., Hosny K.M., Bukhary D.M., Iqbal H., Murshid S.S., Halwani A.A., Alissa M., Menaa F. (2022). Fabrication and characterizations of pharmaceutical emulgel co-loaded with naproxen-eugenol for improved analgesic and anti-inflammatory effects. Gels.

[38] Md S., Alhakamy N.A., Aldawsari H.M., Kotta S., Ahmad J., Akhter S., Shoaib Alam M., Khan M.A., Awan Z., Sivakumar P.M. (2020). Improved Analgesic and Anti-Inflammatory Effect of Diclofenac Sodium by Topical Nanoemulgel: Formulation Development—In Vitro and In Vivo Studies. J. Chem..

[39] Tas C., Ozkan Y., Okyar A., Savaser A. (2007). In vitro and ex vivo permeation studies of etodolac from hydrophilic gels and effect of terpenes as enhancers. Drug Deliv..

[40] Binder L., Mazál J., Petz R., Klang V., Valenta C. (2019). The role of viscosity on skin penetration from cellulose ether-based hydrogels. Ski. Res. Technol..

[41] Bajaj S., Singla D., Sakhuja N. (2012). Stability testing of pharmaceutical products. J. Appl. Pharm. Sci..

[42] Coc L.M.C., Lacatusu I., Badea N., Barbinta-Patrascu M.E., Meghea A. (2021). Effective Lipid Nanocarriers Based on Linseed Oil for Delivery of Natural Polyphenolic Active. J. Nanomater..

[43] Huang J., Wang Q., Li T., Xia N., Xia Q. (2017). Nanostructured lipid carrier (NLC) as a strategy for encapsulation of quercetin and linseed oil: Preparation and in vitro characterization studies. J. Food Eng..

[44] Mao Y., Chen X., Xu B., Shen Y., Ye Z., Chaurasiya B., Liu L., Li Y., Xing X., Chen D. (2019). Eprinomectin nanoemulgel for transdermal delivery against endoparasites and ectoparasites: Preparation, in vitro and in vivo evaluation. Drug Deliv..

[45] Sultan O.S., Kantilal H.K.A.L., Phaik K.S., Choudhury H., Davamani F. (2023). Formulation and Characterization of a Novel Palm-Oil-Based α-Mangostin Nano-Emulsion (PO-AMNE) as an Antimicrobial Endodontic Irrigant: An In Vitro Study. Processes.

[46] Kaur N., Chugh V., Gupta A.K. (2014). Essential fatty acids as functional components of foods—A review. J. Food Sci. Technol..

[47] Jumina J., Mutmainah M., Purwono B., Kurniawan Y.S., Syah Y.M. (2019). Antibacterial and antifungal activity of three monosaccharide monomyristate derivatives. Molecules.

[48] Qasim N., Shahid M., Yousaf F., Riaz M., Anjum F., Faryad M.A., Shabbir R. (2020). Therapeutic Potential of Selected Varieties of *Phoenix dactylifera* L. Against Microbial Biofilm and Free Radical Damage to DNA. Dose-Response.

[49] Ortiz A.C., Yañez O., Salas-Huenuleo E., Morales J.O. (2021). Development of a Nanostructured Lipid Carrier (NLC) by a Low-Energy Method, Comparison of Release Kinetics and Molecular Dynamics Simulation. Pharmaceutics.

[50] Shehata T.M., Elsewedy H.S. (2022). Paclitaxel and Myrrh oil Combination Therapy for Enhancement of Cytotoxicity against Breast Cancer; QbD Approach. Processes.

[51] Haroun M., Elsewedy H.S., Shehata T.M., Tratrat C., Al Dhubiab B.E., Venugopala K.N., Almostafa M.M., Kochkar H., Elnahas H.M. (2022). Significant of injectable brucine PEGylated niosomes in treatment of MDA cancer cells. J. Drug Deliv. Sci. Technol..

[52] Weng J., Tong H.H.Y., Chow S.F. (2020). In Vitro Release Study of the Polymeric Drug Nanoparticles: Development and Validation of a Novel Method. Pharmaceutics.

[53] Souto E.B., Baldim I., Oliveira W.P., Rao R., Yadav N., Gama F.M., Mahant S. (2020). SLN and NLC for topical, dermal, and transdermal drug delivery. Expert Opin. Drug Deliv..

[54] Müller R.H., Hespeler D., Jin N., Pyo S.M. (2019). smartPearls-Novel physically stable amorphous delivery system for poorly soluble dermal actives. Int. J. Pharm..

[55] Andleeb M., Shoaib Khan H.M., Daniyal M. (2021). Development, Characterization and Stability Evaluation of Topical Gel Loaded with Ethosomes Containing *Achillea millefolium* L. Extract. Front. Pharmacol..

[56] Min J.Y., Kim H.J. (2020). Sol–Gel-based Fluorescent Sensor for Measuring pH Values in Acidic Environments. Bull. Korean Chem. Soc..

[57] Ali A., Ali A., Rahman M.A., Warsi M.H., Yusuf M., Alam P. (2022). Development of nanogel loaded with lidocaine for wound-healing: Illustration of improved drug deposition and skin safety analysis. Gels.

[58] Afreen U., Fahelelbom K.M., Shah S.N.H., Ashames A., Almas U., Khan S.A., Yameen M.A., Nisar N., Asad M.H.H.B., Murtaza G. (2022). Formulation and evaluation of niosomes-based chlorpheniramine gel for the treatment of mild to moderate skin allergy. J. Exp. Nanosci..

[59] Abdallah M.H., Lila A.S.A., Unissa R., Elsewedy H.S., Elghamry H.A., Soliman M.S. (2021). Brucine-Loaded Ethosomal Gel: Design, Optimization, and Anti-inflammatory Activity. AAPS PharmSciTech.

[60] Abdallah M.H., Abu Lila A.S., Unissa R., Elsewedy H.S., Elghamry H.A., Soliman M.S. (2021). Preparation, characterization and evaluation of anti-inflammatory and anti-nociceptive effects of brucine-loaded nanoemulgel. Colloids Surf. B Biointerfaces.

[61] Hombach M., Zbinden R., Böttger E.C. (2013). Standardisation of disk diffusion results for antibiotic susceptibility testing using the sirscan automated zone reader. BMC Microbiol..

[62] Balouiri M., Sadiki M., Ibnsouda S.K. (2016). Methods for in vitro evaluating antimicrobial activity: A review. J. Pharm. Anal..

[63] Ávila-Salas F., Marican A., Pinochet S., Carreño G., Valdés O., Venegas B., Donoso W., Cabrera-Barjas G., Vijayakumar S., Durán-Lara E.F. (2019). Film Dressings Based on Hydrogels: Simultaneous and Sustained-Release of Bioactive Compounds with Wound Healing Properties. Pharmaceutics.

