# Supplementary figures and images for: Date Palm Extract (Phoenix dactylifera) Encapsulated into Palm Oil Nanolipid Carrier for Prospective Antibacterial Influence
Source: Plants (Basel). 2023 Oct 25;12(21):3670. doi: 10.3390/plants12213670 (PMC10648499; doi:10.3390/plants12213670)

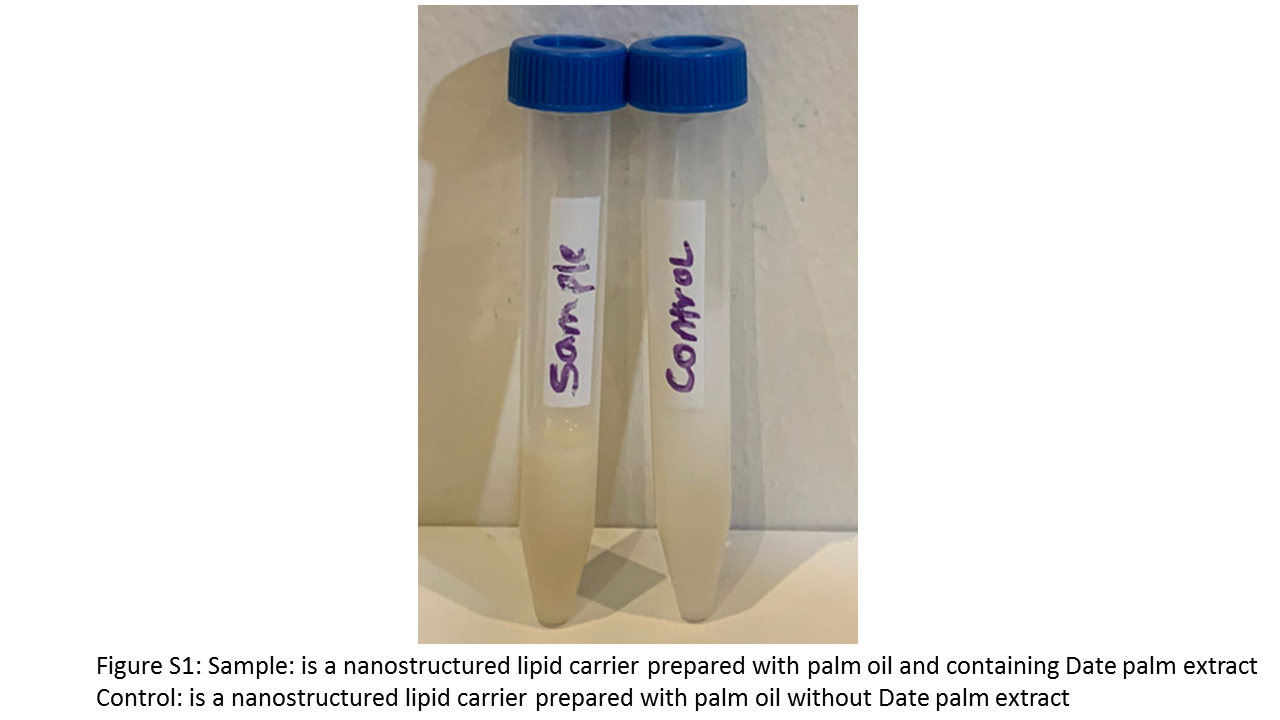

Supplement: Supplementary file 1 [file plants-12-03670-s001.zip › plants-2675630-supplementary.bmp]
